# Biodiversity Governance: A Tower of Babel of Scales and Cultures

**DOI:** 10.1371/journal.pbio.1002108

**Published:** 2015-03-12

**Authors:** Jorge Soberón, A. Townsend Peterson

**Affiliations:** Biodiversity Institute and Department of Ecology and Evolutionary Biology, University of Kansas, Lawrence, Kansas, United States of America

## Abstract

The recently created Intergovernmental Platform on Biodiversity and Ecosystem Services (IPBES), originally focused on multilateral and global issues, is shifting its focus to address local issues and to include in its assessments local stakeholders and indigenous and traditional systems of knowledge. Acknowledging that full biodiversity governance is unavoidably rooted in participation of local actors and their problems and knowledge, we suggest that to deal successfully with the complexity and diversity of local issues, including indigenous knowledge systems, IPBES must recognize a key role of local institutions.

## Abstract 2

To achieve full biodiversity governance requires the participation of local actors and their problems and knowledge, therefore the Intergovernmental Platform on Biodiversity and Ecosystem Services (IPBES) must recognize a key role of local institutions.

## Introduction

In April 2011, 90 governments established the Intergovernmental Platform on Biodiversity and Ecosystem Services (IPBES; http://www.ipbes.net/), with the principal objective of responding to “…requests for scientific information related to biodiversity and ecosystem services from governments, relevant multilateral environmental agreements, and United Nations bodies, as well as other relevant stakeholders” [1]. This undertaking is ambitious, and several authors have discussed perils that IPBES will face: the need for transparency [2], avoiding stretching too much the weak analogy between IPBES and the Intergovernmental Panel on Climate Change (IPCC) [3,4], and underestimating the nonlinear and fluid features of biodiversity governance [5]. Here, we point out yet another risk for IPBES: attempting to shift the focus away from its natural place, global scales of biodiversity governance, and implying that IPBES can also be relevant at national or local levels, or promising to include indigenous and local knowledge (ILK) [6,7].

IPBES was envisioned as managing “the assessment component” of the science policy landscape for the Convention on Biological Diversity (CBD) and other environmental multilateral agreements [8]. This task implies a large-scale scope of work. However, IPBES is now considering “conceptual frameworks” (CFs) that will provide “a common analytical basis … for all IPBES stakeholders” [7]. CFs appear to be intended to be applied at fine levels in the governance hierarchy [9]. The document “Revised draft stakeholder engagement strategy” (still being reviewed by IPBES) explicitly mentions as stakeholders:

… *community members*, *indigenous people*, *members of non-governmental organizations and community-based organizations*, *business people*, *farmers*, *local governments*, *intergovernmental organizations and a multitude of other organizations and individuals involved in implementation ofbiodiversity and ecosystem services work on the ground*.

The above are clear signals that IPBES intends to operate at levels finer than the global or include ILKs in global assessments. Several risks are involved in trying to cover many scales using the modus operandi of a multilateral organization, as we describe below. The point of this communication is that IPBES should use a language that recognizes much more explicitly the complexities of working across multiple scales and the need to include local institutions.

## Multiplicity of Biodiversity Governance Structures

It is almost an axiom of biodiversity governance that its features change with level of focus [10–13]. The question is not whether multiscale work is required (it is), but whether a CF described as a Rosetta Stone for biodiversity concepts [9] is the right solution to address these shifting scales. The features of biodiversity governance change across scales (local, subnational, national, regional, and global) because the scale determines the identity of the stakeholders and therefore the questions and themes of interest. Changing scales determines the nature and availability of the knowledge that may be used as a base for decisions and the way in which decisions are taken [14].

An IPBES focusing at global and regional scales, as originally conceived, appears feasible and useful [15]. Indeed, at a global level, the themes and actors to engage in biodiversity governance are manageably few in number, as discussed by van den Hove and Chabason [15] based on the gap analysis commissioned by the IPBES Governing Body [16]. The topics and questions raised by such stakeholders relate mostly to the interests of the multilateral environmental agreements. Conflictive and difficult as negotiations at this level may be, the rules are basically shared and understood by most global stakeholders. By focusing at this level, IPBES may hope to minimize institutional mismatches [5], wherein the nature of a governance problem and the institutional arrangements established to address it are misaligned. Moreover, the amount of data available for assessments at a global level is also very significant: for example, the volume of global-scope, remote-sensing data is measured in petabytes, it keeps growing, and it is widely available [17]. The amount of raw data about species’ occurrences is reaching a terabyte, grows daily, and is largely available through the portal of the Global Biodiversity Information Facility (GBIF) [18]. Also at global extents and coarse resolutions, the International Union for Conservation of Nature (IUCN) and BirdLife International serve hundreds of megabytes of digital maps of distributions of terrestrial vertebrates of the world. The number of global-level reports and analyses about biodiversity and ecosystem function is simply too large to be reviewed here.

All the above suggests that an IPBES focused at global levels, using data and tools already available, would be able to deal with many assessment aspects of relevant questions at broad scales, responding to a reasonable number of important stakeholders, within a well-understood (if difficult) governance process. However, if IPBES instead tries to address such aspects in the biodiversity agenda that include local stakeholders, at national and local scales, and involving other knowledge systems, it will face complicated and expensive challenges.

When the scale moves to the country level, or further still, to more local extents, the details of the biodiversity agendas become overwhelmingly important, in all their ecological, economic, social, and cultural diversity, and for thousands of specific and mostly unique cases. Governance processes at local levels are complex, varied, and, if not already in place, require much time and effort to be established and accepted. The gap analysis commissioned by the Government Council of IPBES recognized the existence and complexity of such manifold science policy interfaces [16]. Moreover, at local levels, scientific data and information are generally very scarce [19], and obtaining them de novo may be impossible or very slow, or extremely expensive [20]. Most IPBES documents pay only lip service to the problem of local data scarcity, ignore it, or (increasingly), assume that existing local ILKs can be tapped to fill gaps. If IPBES, as such, with its government-dominated, multilateral modus operandi, tries to engage successfully at local levels, it will face a myriad of intractable problems. The treatment of indigenous knowledge in the current CF [21] is a good example to the point.

## Indigenous Knowledge

No doubt exists about the depth and richness of knowledge that the different peoples of the world have developed and maintained over history [22], or that this knowledge can be used valuably to deal with many environmental problems, mostly of local nature (for many examples see http://www.equatorinitiative.org/). However, what is risky is to assume that: (1) assessments at global or regional scales can successfully include a variety of indigenous groups and make use of their knowledge in ways that serve IPBES’ goals; or (2) that IPBES can expect to influence successfully the way that hundreds or thousands of locally relevant assessments will be conducted, resorting to a general scheme represented by CFs.

Communicating across diverse cultural paradigms using the very linear and formalized structures of IPBES [5] is unlikely to work. IPBES’ own document about principles and procedures for working with indigenous and local peoples [23] includes caveats and warnings about the lack of harmony between IPBES’ formalized diplomatic structures and the ideal formats to work with traditional peoples. Coexistence and mutual fertilization of western science and ILKs, although possible, is never easy [24–26]; indeed, unless the process is conducted with great respect, inclusiveness, and care, holders of ILKs tend to experience inequities and power differences vis a vis western scientists and their institutions [23,26–28]. It is also the case that “representatives” of indigenous peoples may not represent accurately (even with the best of intentions) the actual position of entire communities [29]. Therefore, if the above caveats are taken seriously, it is difficult to see how IPBES can realistically and appropriately engage with one, much less with many, indigenous groups, in any large-scale assessment. When such is attempted, as for instance for the Pollinators Assessment (http://www.unesco.org/new/en/links_call), IPBES’ own guidelines of how to engage with indigenous groups [23] should be followed strictly.

However, at national or local levels, when the appropriate stakeholders decide to launch their own assessments, indigenous communities will often be prominent stakeholders; including their participation, and maybe their knowledge, will be fair and indispensable. In these cases, the role of IPBES should be at most one of advisor, since it hardly has the data, resources, or credibility (at local levels) to deal with the intricacies of local governance processes. In this respect, it may well be the case that it is IPBES’ detailed document on approaching indigenous communities [23] that may be most useful, rather than the CF, where ILK is treated in what we think is a misleading way.

This point is well-illustrated in Fig 1 of the CF [7]. Twice, “Mother Earth” appears there, as a successful example of incorporating ILK. Díaz et al. [9] state “… the CF goes further than any previous initiative in the international environmental science/policy interface in its explicit, formal incorporation of knowledge systems other than western science…” and then mention approvingly the inclusion of the Mother Earth concept. However, an idea of Mother Earth is not shared as “knowledge” in every culture; rather, it is regarded as part of the “worldview” [23], or “Kosmos,” [22,30] of some. In many ILK systems, the spiritual, the factual, and the practical are deeply united [24,26,31], and trying to disentangle them may be a mistake. However, coincidence of statements of fact among different traditions has been illustrated many times (factual coincidence is what permits the very idea of bioprospecting for medicinal plants [32]), whereas bridging between worldviews is generally much more difficult [23]. By not making this important distinction, the CF is simplifying drastically the complexities of ILKs and assuming that it is already including ILKs, which is debatable. This error is bound to happen every time that indigenous groups are not heard directly, preferably in their own environments, and exerting the enormous effort required to establish trust [23,33]. It is in local assessments, driven by national or local institutions, according to their culture, and with full participation of local stakeholders, that the full complexity of ILKs may have a chance to be expressed.

To summarize, selecting the correct scale at which IPBES should operate may be its most crucial decision. IPBES, understood as a multigovernmental platform operating by the rules and procedures of the United Nations, started at the correct level (the global), but is being dragged into more local and inclusive scopes by its own acknowledgment of the fact that local governance is indispensable in dealing comprehensively with biodiversity issues. The risk lies in assuming that a single international organization can or should deal with every level in the hierarchy of biodiversity governance. Repeatedly using language that suggests that it can, or worse, attempting to do so ignoring its own warnings and caveats, will gravely endanger its crucial mission. When assessments move away from global levels, local organizations, institutions, and stakeholders will have to assume a dominant role, according to their own circumstances, culture, languages, personnel, and data availability. If a single CF will provide some sort of Rosetta Stone, as described by Diaz et al. [9], the problem of full and engaged participation of local stakeholders, using local data and local governance processes, will have to be addressed every time. An alternative is that IPBES assumes in full the economic, cultural, and informational implications of the multiscale structure of biodiversity governance, use language that explicitly acknowledges the role of national and local institutions to conduct national and local assessments, and resorts to these partners, as available, to fulfill its mandate.

## Abbreviations

CBD: Convention on Biological Diversity
CF: conceptual framework
GBIF: Global Biodiversity Information Facility
ILK: indigenous local knowledge
IPBES: Intergovernmental Platform on Biodiversity and Ecosystem Services
IPCC: Intergovernmental Panel on Climate Change
IUCN: International Union for Conservation of Nature
